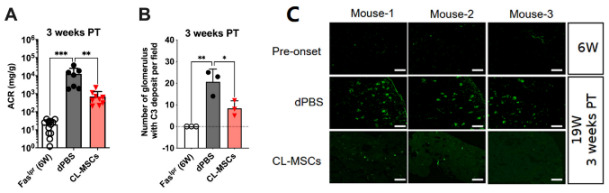# Correction: Chua et al. Intraperitoneally Delivered Umbilical Cord Lining Mesenchymal Stromal Cells Improve Survival and Kidney Function in Murine Lupus via Myeloid Pathway Targeting. *Int. J. Mol. Sci.* 2023, *24*, 365

**DOI:** 10.3390/ijms26051997

**Published:** 2025-02-25

**Authors:** Alvin Wen Choong Chua, Dianyang Guo, Jia Chi Tan, Frances Ting Wei Lim, Chee Tian Ong, Jeyakumar Masilamani, Tony Kiat Hon Lim, William Ying Khee Hwang, Ivor Jiun Lim, Jinmiao Chen, Toan Thang Phan, Xiubo Fan

**Affiliations:** 1 Department of Plastic, Reconstructive and Aesthetic Surgery, Singapore General Hospital, Singapore 169856, Singapore; alvin.chua.w.c@singhealth.com.sg; 2 Department of Clinical Translational Research, Singapore General Hospital, Singapore 169608, Singapore; guo.dianyang@sgh.com.sg (D.G.); frances.lim.t.w@sgh.com.sg (F.T.W.L.); 3 Single-Cell Computational Immunology, Singapore Immunology Network, Singapore 138668, Singapore; e0546189@u.nus.edu (J.C.T.); chen_jinmiao@immunol.a-star.edu.sg (J.C.); 4 CellResearch Corporation Pte Ltd., Singapore 048943, Singapore; ongcheetian@cordlabs.sg (C.T.O.); jeyakumar@cordlabs.sg (J.M.); ivorlim@cellresearchcorp.com (I.J.L.); 5 Department of Anatomical Pathology, Singapore General Hospital, Singapore 169856, Singapore; lim.kiat.hon@singhealth.com.sg; 6 Department of Hematology, Singapore General Hospital, Singapore 169856, Singapore; william.hwang.y.k@singhealth.com.sg; 7 National Cancer Centre Singapore, Singapore 169610, Singapore; 8 Department of Surgery, Yong Loo Lin School of Medicine, National University of Singapore, Singapore 119228, Singapore; 9 SingHealth Duke-NUS Medicine Academic Clinical Programme, Duke-NUS Medical School, Singapore 169857, Singapore

## Error in Figure

In the original publication, there was a mistake in Figure 2C as published [1]. The image for Mouse-1 in dPBS group was also used as the image for Mouse-2 in the dPBS group. The corrected Figure 2C appears below. The authors state that the scientific conclusions are unaffected. This correction was approved by the Academic Editor. The original publication has also been updated.